# Influence of drying time and sugar content on the sensory profile of beef jerky

**DOI:** 10.1038/s41538-025-00433-8

**Published:** 2025-05-05

**Authors:** Vitor Andre Silva Vidal, Rikke Harveland Ølberg, Lene Waldenstrøm, Ida-Johanne Jensen, Jørgen Lerfall

**Affiliations:** Department of Biotechnology and Food Science, NTNU-Norwegian University of Science and Technology, NO-7491 Trondheim, Norway

**Keywords:** Nutrition, Agriculture, Science, technology and society

## Abstract

Beef jerky is widely consumed for its convenience, sensory appeal, and shelf stability. Reducing sugar content is a public health priority but presents technological and sensory challenges. Drying time also plays a crucial role due to its economic and sustainability impacts. This study evaluated the effects of different drying times (2, 3, 4, and 6 h) and sugar levels (0.5%, 1%, and 1.5%) on beef jerky’s sensory and physicochemical properties. Drying time significantly influenced product quality, affecting sensory attributes. Results showed that reducing added sugar did not compromise the sensory properties preferred by consumers. Moreover, optimizing both drying time and sugar content can improve production efficiency while maintaining consumer acceptance. These findings highlight the potential for healthier beef jerky with enhanced sustainability.

## Introduction

Beef jerky is a popular dried meat product consumed worldwide, possessing an excellent nutritional profile, unique sensory properties, and offering convenience due to its non-refrigeration requirement and shelf stability^1,2^.

The traditional preparation process of beef jerky involves cutting, marinating, and drying, and these processing steps contribute to the final quality of the product^3^. The drying process is a crucial operation in the food industry, essential for prolonging shelf life by preventing microbial growth and enzymatic activity^4^. Drying parameters such as time, temperature, etc. are easily controlled, making the drying process stable and uniform. The drying can directly affect moisture migration, leading to alterations in the quality of beef jerky, including changes in color and texture properties^5^, and potentially impacting consumer sensory acceptance. Hence, a better understanding of the drying process could enhance the efficiency of beef jerky production.

Beef jerky typically has a low water activity (around 0.70–0.85) and a moisture-to-protein ratio of less than 0.75, resulting in a dry and firm product^1^. Meat tenderness is closely linked to consumer satisfaction, and improving the texture characteristics of beef jerky is a high priority in the meat industry^6–8^ to meet the growing consumer demand for high-quality products^9^.

In the beef jerky processing, the curing solution includes water, sugar, salt (NaCl), sodium nitrite (NaNO_2_), and spices^4,10,11^. Excessive sugar intake is currently a major concern in the fields of nutrition and health due to its harmful effects on consumer health^12^. Studies have demonstrated that high consumption of free sugars is linked to several health disorders, including obesity, dyslipidemia, type 2 diabetes, dental caries, metabolic syndrome, cardiovascular disorders, and fatty liver disease^13–15^. Moreover, excessive intake of free sugars can affect neural systems, alter emotional processing, and contribute to depression and anxiety^16^. Furthermore, consumers are becoming more aware of the sugar and calorie content in foods^17^. Consequently, the high sugar content in foods is a concern, and reformulating these products to be healthier presents a significant challenge. The effect of drying time has been well studied previously; however, the combination of different drying times and sugar content levels and their effects on sensory properties of beef jerky has not been adequately investigated.

Considering all this, the objective of this study was to evaluate the effects of different drying times (2, 3, 4, and 6 h) and sugar content added (0.5%, 1%, and 1.5%) on the sensory properties and physicochemical characteristics of beef jerky.

## Results and discussion

### Water activity

**Fig. 1:**
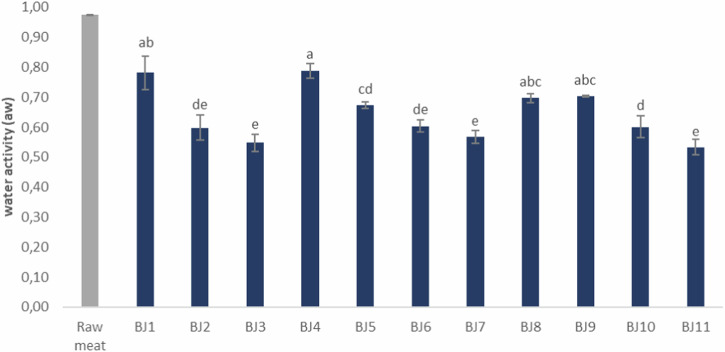
Water activity of beef jerky prepared with different protocols. Values are presented as means. The error bars represent the standard error. Different letters represent statistically significant difference by the Tukey test (*P* < 0.05). BJ5 and BJ7: 0.5% sugar. BJ9 and BJ11: 1.5% sugar. B5 and B9: 3 hours drying. B7 and B11: 6 hours drying.

Water activity (a_w_) is a critical factor in food safety, where lower a_w_ correlates with extended shelf life. As expected, a significant gradual decrease (*P* < *0.05*) in a_w_ was observed during the drying process of the beef jerky samples (Fig. 1).

Beef jerky samples dried for 6 h (BJ3, BJ7, and BJ11) had the lowest (*P* < *0.05*) a_w_ compared to the other samples. On the other hand, there was no significant difference (*P* > *0.05*) between samples subjected to the same drying time despite containing different sugar concentrations (0.5, 1, or 1.5%). Thus, the sugar content did not affect either the drying process or the a_w_ of the beef jerky samples.

An acceptable limit for a_w_ in a shelf-stable product is often defined as <0.85, a value sufficient to prevent bacterial growth in vacuum-packed products stored at room temperature^18,19^. However, several studies indicate that for a shelf life exceeding 60 days at room temperature, a_w_ should be <0.75 to ensure microbial stability^4,20–23^. Since the samples that were dried for 2 h resulted in higher aw values, they were excluded from further sensory evaluation.

The drying time during beef jerky processing significantly affected the quality of the final product. As expected, longer drying times significantly reduced the a_w_ and moisture content values. Drying for only 2 h may decrease shelf stability due to an a_w_ of approximately 0.8.

### Texture profile

Texture parameters play a crucial role in consumer acceptance^24^ and are associated with water retention capacity^25^. Comprehending the effects of processing on texture profiles is therefore essential for optimizing production and enhancing the final product quality^26^, especially in tough dried meat products such as jerky^11,27^.

The texture properties of beef jerky are directly related to moisture content and moisture content positively affects the tenderness and juiciness characteristics of the beef jerky samples. However, a high moisture content will often result in a higher a_w_, thereby reducing shelf life^7^.

The results of the texture properties for beef jerky samples treated with varying levels of added sugar and drying times are presented in Table 1. The beef jerky samples subjected to a 2-hour drying process (BJ1, BJ4, and BJ8), exhibited the highest values (*P* < *0.05*) for hardness, cohesiveness, gumminess, and chewiness compared to the samples dried for 4 and 6 h. The samples with lower added sugar (BJ4, BJ5, BJ6, and BJ7) tended to show lower values for hardness, cohesiveness, gumminess, and chewiness. However, it is not statistically significant (*P* > *0.05*).

**Table 1:** Texture profile of beef jerky (BJ) prepared with different protocols

Sample	Hardness (*N*)	Cohesiveness	Gumminess	Chewiness (*N*)
**Raw meat**	4.50 ± 1.1	0.63 ± 0.02	2.76 ± 0.65	2.38 ± 0.58
BJ1	109.03 ± 4.82^a^	0.86 ± 0.30^a^	93.72 ± 4.75^a^	88.07 ± 4.87^a^
BJ2	67.98 ± 4.47^bcd^	0.65 ± 0.03^cde^	44.58 ± 3.61^cd^	31.44 ± 2.89^cd^
BJ3	81.18 ± 7.03^abc^	0.68 ± 0.04^cde^	56.46 ± 7.69^bc^	47.77 ± 8.47^bcd^
BJ4	100.51 ± 12.60^ab^	0.80 ± 0.02^abc^	81.92 ± 12.31^ab^	73.10 ± 13.04^ab^
BJ5	84.75 ± 6.73^abc^	0.73 ± 0.04^abcde^	62.02 ± 5.71^abc^	48.25 ± 5.38^bcd^
BJ6	70.59 ± 6.08^bcd^	0.69 ± 0.04^cde^	48.83 ± 6.17^cd^	41.87 ± 6.61^bcd^
BJ7	66.68 ± 7.21^bcd^	0.64 ± 0.04^cd^	42.26 ± 5.13^cd^	33.86 ± 4.33^cd^
BJ8	108.39 ± 5.31^a^	0.85 ± 0.01^ab^	92.80 ± 5.02^a^	81.10 ± 3.71^a^
BJ9	65.05 ± 7.07^cd^	0.75 ± 0.05^abcd^	49.44 ± 7.28^cd^	42.51 ± 7.23^bcd^
BJ10	46.39 ± 4.80^d^	0.59 ± 0.03^d^	24.04 ± 3.18^d^	19.21 ± 2.74^d^
BJ11	89.54 ± 10.79^abc^	0.71 ± 0.03^bcde^	64.58 ± 9.68^abc^	56.35 ± 9.13^abc^

Values are means ± standard error. Different superscript letters (^abcde^) in the same column represent statistically significant difference by the Tukey test (*P* < *0.05*). BJ1, BJ2, and BJ3: 1% sugar. BJ4, BJ5, B6, and BJ7: 0.5% sugar. BJ8, BJ9, B10, and BJ11: 1.5% sugar. BJ1, BJ4, and BJ8: 2 h drying. BJ5, and BJ9: 3 hours drying. BJ2, BJ6, and B10: 4 hours drying. BJ3, BH7, and BJ11: 6 h drying.

Changes in texture profile during meat processing result from complex chemical transformations in muscle and connective tissue fibers^28,29^. According to Zeng, et al.^30^, the development of texture properties primarily results from the acid-induced gelation of muscle proteins and the quantity of water released. Davey and Gilbert^31^ documented the variation in texture values due to temperature and the effect of heat-induced denaturation of meat proteins. Heating meat leads to increased toughness, attributed to myofibrillar denaturation^32^. Enhanced meat tenderness is associated with collagen denaturation^33^, and increased proteolytic activity also contributes to meat tenderization^29^.

Considering the results of the texture profile of beef jerky samples, it can be observed that longer drying times reduce hardness, cohesiveness, gumminess, and chewiness. Similar results were found by Bulgaru, et al.^29^, who studied the texture profile of beef during the dry-ageing process. According to several authors^34–36^, moisture content affects the texture properties of beef jerky samples.

### Sensory evaluation and characterization

To select beef jerky samples for sensory evaluation, a_w_, texture profile, and sensory attributes (assessed by a semi-trained panel of 10 individuals) were evaluated for the 11 different beef jerky protocols using different sugar levels (0.5%, 1%, and 1.5%) and drying times (2, 3, 4, and 6 h). As mentioned, the samples subjected to 2 h of drying (BJ1, BJ4, and BJ8) had a_w_ > 0.75, making them undesirable for long-term storage at room temperature due to microbiological risks^20–23^.

Based on the results presented in Section *2.1 Water Activity*, *2.2 Texture Profile*, and the sensory attribute evaluation conducted by the semi-trained panel as explained in section *2.5 Sensory Evaluation*, the samples BJ5, BJ7, BJ9, and BJ11 were selected for sensory evaluation (acceptance, CATA questionnaire, and ideal product analysis). The selection also considered the greatest difference in added sugar content (0.5% and 1.5%).

By reducing the number of samples for sensory analyses to four, the potential impacts on sensory test results were also reduced. Testing a large number of samples or sample sets can lead to sensory adaptation, resulting in decreased sensory sensitivity^37^.

A proximate composition analysis was conducted to characterize the samples that were sensory evaluated (Table 2). The beef jerky samples subjected to the same drying time exhibited differences (*P* < *0.05*) in proximate composition, with moisture content ranging from 11% to 21.1%, ash content from 4.9% to 6.0%, protein content from 61.1% to 68.1%, and lipid content from 9.2% to 13.9%.

**Table 2:** Proximate composition of beef jerky prepared with different protocols

Sample	Moisture (%)	Ash (%)	Protein (%)	Lipid (%)
BJ5	17.69 ± 0.07^b^	6.02 ± 0.04^a^	67.99 ± 0.34^a^	9.18 ± 0.46^b^
BJ7	11.72 ± 0.03^c^	5.56 ± 0.04^b^	68.06 ± 0.08^a^	12.28 ± 0.67^a^
BJ9	21.14 ± 0.07^a^	4.87 ± 0.06 ^d^	61.08 ± 0.63^b^	9.17 ± 0.49^b^
BJ11	10.99 ± 0.08^c^	5.24 ± 0.05^c^	67.51 ± 0.58^a^	13.91 ± 0.88^a^

Values are means ± standard error. Means in the same column with different superscript letters (^abc^) are significantly different by the Tukey test (*P* < *0.05*). BJ5 and BJ7: 0.5% sugar. BJ9 and BJ11: 1.5% sugar. BJ5 and BJ9: 3 hours drying. BJ7 and BJ11: 6 h drying.

As expected, the samples subjected to longer drying times (BJ7 and BJ11) had the lowest moisture content and the highest lipid content compared to the other samples (BJ5 and BJ9). To achieve microbial safety and desirable texture properties in beef jerky products, it is recommended that the moisture content be reduced to around 20% and water activities maintained below 0.8^38^. All samples exhibited protein content greater than 61%, indicating they are a promising source of protein for quick and easy consumption. Typically, commercially available ready-to-eat sliced jerky products have a protein content greater than 45%^4^.

The variations and significant differences among the samples in terms of ash and protein content are likely due to the typical heterogeneity of beef jerky samples, resulting from the different compositions of the meat used, even though they were obtained from the same supplier and used the same meat cut.

The results of the CATA questionnaire are presented in Table 3. A significant difference (*P* < *0.05*) between the samples was observed in 7 sensory descriptors: red and brown color, astringent, chewy, crispy, tender, and juicy.

**Table 3:** Results of CATA (check all that apply) questionnaire concerning sensory descriptors (aroma, appearance, taste, and texture characteristics) of beef jerky samples

Descriptors	BJ5	Sample		
		BJ7	BJ9	BJ11
**Aroma**				
Smoke	38^a^	44^a^	34^a^	31^a^
Meat	51^a^	43^a^	56^a^	55^a^
Rancid	10^a^	14^a^	10^a^	12^a^
**Appearance**				
Dark red	55^a^	48^a^	54^a^	51^a^
Red	19^a^	10^b^	27^a^	13^b^
Brown	16^b^	34^a^	19^b^	27^a^
Dry appearance	49^a^	51^a^	44^a^	44^a^
**Taste**				
Smoked meat	54^a^	48^a^	49^a^	51^a^
Aftertaste	26^a^	18^a^	25^a^	16^a^
Umami	25^a^	26^a^	28^a^	26^a^
Salty	39^a^	36^a^	25^a^	31^a^
Sweet	14^a^	8^a^	17^a^	15^a^
Pepper	12^a^	21^a^	18^a^	13^a^
Bitter	1^a^	5^a^	3^a^	5^a^
Astringent	13^b^	30^a^	16^b^	20^ab^
Bland	29^a^	26^a^	35^a^	33^a^
**Texture**				
Chewy	41^a^	14^b^	36^a^	13^b^
Crispy	10^b^	54^a^	13^b^	52^a^
Hard	29^a^	38^a^	23^a^	34^a^
Dry	48^a^	59^a^	47^a^	53^a^
Fibrous	23^a^	31^a^	26^a^	21^a^
Tender	18^a^	6^b^	22^a^	11^ab^
Juicy	18^a^	3^b^	15^a^	8^ab^

Values in the same row with different superscript letters (^ab^) are significantly different by the Cochran’s Q test and McNemar’s test (*P* < *0.05*). BJ5 and BJ7: 0.5% sugar. BJ9 and BJ11: 1.5% sugar. BJ5 and BJ9: 3 hours drying. BJ7 and BJ9: 6 hours drying.

Consumers identified significant differences (*P* < *0.05*) primarily in texture attributes (chewy, crispy, tender, and juicy) among the beef jerky samples. Additionally, participants did not perceive differences between samples based on the added sugar content (0.5% or 1.5%) during processing. However, varying drying times (3 or 6 h) significantly influenced the results (*P* < *0.05*).

Participants found the samples subjected to 3 h of drying (BJ5, BJ9) to be significantly (*P* < *0.05*) chewier and tender compared to samples dried for 6 h (BJ7, BJ11). Furthermore, samples dried for 6 h were significantly (*P* < *0.05*) crispier than those dried for 3 h.

Samples dried for 6 h (BJ7, BJ11) were significantly (*P* < *0.05*) browner, while those dried for 3 h (BJ5, BJ9) were significantly (*P* < *0.05*) redder. According to Shi, et al. ^5^, who evaluated beef jerky samples prepared with different drying times and temperatures, the effect of drying time on the appearance of the samples was significant at the same drying temperature.

**Fig. 2:**
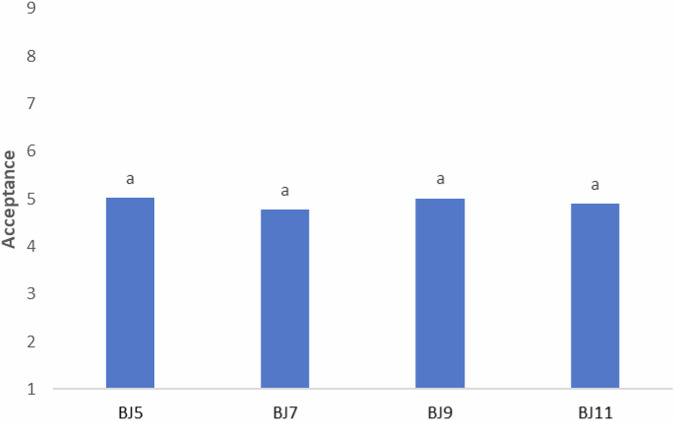
Acceptance values of beef jerky treatments. The participants rated the samples for overall liking using a 9-point structured hedonic scale (1 – extremely disliked to 9 – extremely liked). Values are means (*n* = 93). The superscript letter (a) indicates no statistically significant difference according to Tukey’s test (*P* < 0.05). BJ5 and BJ7: 0.5% sugar. BJ9 and BJ11: 1.5% sugar. B5 and B9: 3 hours drying. B7 and B11: 6 hours drying.

The results of the acceptance test can be seen in Fig. 2. The mean acceptance scores were considered neutral for all samples, ranging from 4.77 to 5.02, with no significant difference (*P* > *0.05*).

This suggests that reducing the added sugar content and drying time during processing is possible without affecting the final product’s acceptance. These results are significant due to the importance of reducing sugar consumption to make foods healthier, especially in meat products with high sodium content like beef jerky. Additionally, reducing drying time can increase time and cost efficiency in the food industry.

**Fig. 3:**
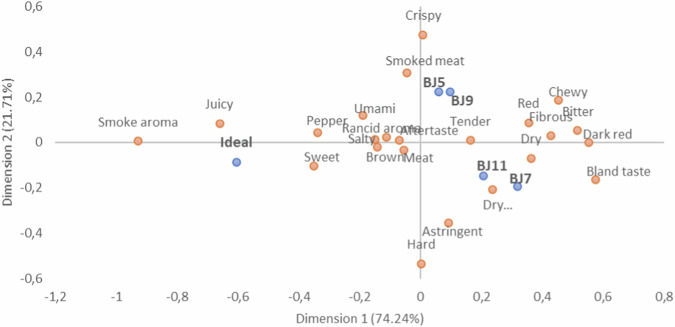
Representation (*n* = 93) of the samples, ideal product, and the sensory attributes in the first and second dimensions of the correspondence analysis on data questions check-all-that-apply (CATA) of selected beef jerky treatments. BJ5 and BJ7: 0.5% sugar. BJ9 and BJ11: 1.5% sugar. B5 and B9: 3 h drying. B7 and B11: 6 h drying.

During the sensory evaluation, the panelists were asked to select which sensory attributes in beef jerky samples they desired in an ideal product. To map these desired sensory characteristics for an ideal product, Correspondence Analysis (CA) was conducted including the ideal product, as shown in Fig. 3.

**Fig. 4:**
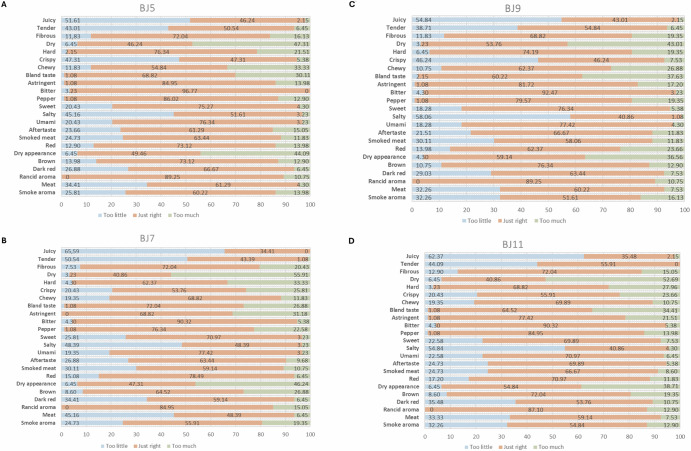
Just About Right (JAR) results of sensory attributes of beef jerky samples (n = 93). **A**–**D** BJ5 and BJ7: 0.5% sugar. BJ9 and BJ11: 1.5% sugar. B5 and B9: 3 hours drying. B7 and B11: 6 h drying.

The CA plot had a cumulative dimension value of 95.95%, effectively describing the relationship between attributes and products in two dimensions^39^. Additionally, JAR was performed to evaluate the acceptance of the different sensory attributes in relation to consumer preferences for sensory attributes in an ideal product, as illustrated in Fig. 4. The JAR results were interpreted with the CA plot results, including the position of the ideal product, to gain a comprehensive understanding of consumer perceptions of an ideal beef jerky.

Consistent opinions about the attributes of the samples compared to the description of the ideal beef jerky product by consumers, as shown in Fig. 4, indicate that the samples evaluated were insufficiently juicy, salty, dark, and lacked meat flavor. Additionally, the samples were considered too dry and had a dry appearance. The samples subjected to 3 h of drying (BJ5, and BJ9) had low crispiness, while those dried for 6 h were not sufficiently dark.

Evaluating the positions of the samples on the CA plot (Fig. 3), it was mainly the drying times that influenced the attributes. Another important finding was that the added sugar content during processing did not significantly affect the attributes, as samples with different sugar contents (0.5% and 1.5%) but the same drying time grouped close to each other. Therefore, it can be concluded that it is possible to reduce the added sugar content in beef jerky samples without significantly affecting sensory attributes, which is considered ideal for the final product.

The ideal product and JAR results demonstrated that reducing the added sugar content is possible without affecting the sensory attributes considered ideal by consumers. Additionally, samples using the same drying time exhibited similar sensory characteristics.

## Methods

### Material

Eleven beef loin (*Longissimus lumborum*) was used for each replication of the experiment. The cut was purchased from slaughterhouses (Trondheim, Norway) with assured hygienic quality. Sodium nitrite was purchased from Merck (Germany). Salt and sugar were purchased from a local grocery market (Trondheim, Norway).

### Protocols

The beef jerky samples were prepared according to Yang, et al.^40^ with some modifications to evaluate the effects of drying time (ranging from 2 to 6 h) and sugar content (ranging from 0.5 to 1.5%). The samples are detailed in Table 4.

**Table 4:** Beef jerky prepared with different sugar content and drying time

Sample	Sugar content (%)	Drying time (hours)
BJ1	1	2
BJ2	1	4
BJ3	1	6
BJ4	0.5	2
BJ5	0.5	3
BJ6	0.5	4
BJ7	0.5	6
BJ8	1.5	2
BJ9	1.5	3
BJ10	1.5	4
BJ11	1.5	6

To avoid an excess of samples for sensory evaluation by consumers and the consequent influence on the answers, four treatments (BJ5, BJ7, BJ9, BJ11) were selected for sensory evaluation based on the results from the texture profile, water activity (a_w_), and Grid method^41,42^. The methods are described in the following topics.

### Processing

The beef loin (*Longissimus lumborum*) was sliced using a meat slicer to a thickness of 5 mm (Compact 1 RIS518010 Ritter, Germany). Subsequently, the slices underwent a salting step for 18 h at 3 °C using 70% water, 1.5% NaCl, and 150 ppm NaNO_2_ based on the weight of the meat, varying only the sugar content (Table 4). After the salting step, the samples were smoked in a smoking chamber (CS700 EL MAXI 1001, Germany) at 30 °C for two hours, and at 70 °C for one hour using beechwood chips (RÄUCHERGOLD HBK 750-2000 Delikatess-Räucherspäne Beechwood Chips). After smoking step, the drying process was conducted, varying from 2 to 6 h (70 °C) depending on the sample (Table 4). The final product was cooled to room temperature and vacuum-packed in polyethylene packages.

Three independent processes performing all samples were conducted at the Department of Biotechnology and Food Science at the Norwegian University of Science and Technology, Trondheim, Norway.

### Physicochemical characterization

The moisture, protein, lipids, and ash contents in the beef jerky samples were determined according to Horwitz^43^. The water activity (a_w_) was assessed at 20 °C utilizing the LabMaster apparatus (Novasina, Switzerland).

The texture profile was determined using the TA-XT2i texture analyzer (SMS Ltd., Surrey, England). The texture parameters hardness (N/cm^2^), cohesiveness, gumminess, and chewiness (N/cm) were determined according to Luo, et al.^34^. The samples were cut into 1 cm × 1 cm × 1 cm and double compressed to 40% of the original thickness at a test speed of 2 mm/s using a 35 mm diameter stainless steel probe (P-35), with a 5 second delay between compressions. All measurements were performed at room temperature.

### Sensory evaluation

All sensory analyses were conducted in accordance with the Principles of Good Practice^44^. Participation was voluntary, and all participants read and approved the consent form before tasting the samples. All samples were tasted by all participants, and the sensory analyses were conducted in a single session. The samples cut into into 2 cm × 2 cm × 2 cm pieces for sensory analysis were served in identical plastic bowls marked with anonymous three-digit codes for sample identification, randomized for each analysis, and the panelists received one sample in each bowl. The serving order of the samples was balanced to prevent the carry-over effect on the results^44^. The panelists were provided with room-temperature water and a discard cup. The sensory analyses were conducted in the sensory laboratory, a room designed in accordance with the ISO standard “General guidance for the design of test rooms” ISO 8589:2007 at the Department of Biotechnology and Food Science at the Norwegian University of Science and Technology, Trondheim, Norway.

First, a semi-trained panel consisting of 10 assessors (aged between 20 and 49 years), with prior experience and familiarity with meat products^45^, was employed to generate the sensory profile of the samples. The repertory Grid method^41,42^ was applied to identify and describe the similarities and differences in the attributes of appearance, aroma, flavor, and texture of the samples. This was followed by a group discussion to select the most frequently cited descriptors that best characterize the product and to discard those not perceived by most assessors. The selected descriptors were: smoke aroma, meat aroma, rancid aroma, dark red, red, brown, dry appearance, chewy, crispy, hard, dry, fibrous, tender, juicy, smoked meat, umami, salty, sweet, astringent, bland taste, pepper, bitter, and aftertaste. The selected descriptors were used as check options for the subsequent check-all-that-apply (CATA) questionnaire^46^.

Secondly, on the same day and during the same session, a consumer panel consisting of 93 participants, including students and employees of the Norwegian University of Science and Technology in Trondheim, Norway, conducted a CATA questionnaire combined with acceptance on the beef jerky samples. The acceptance was rated using a 9-point structured hedonic scale (1 – extremely disliked to 9 – extremely liked)^47^ followed by the CATA questionnaire where the participants selected the attributes that they believed best described the samples^46^. Finally, the consumers selected the attributes that best described the ideal product^47^.

### Statistical analysis

Three independent processes in different days were conducted using the same methodology, formulation, and technology. For each process replication, a minimum of three samples was collected for each analysis. The results were expressed as the mean values from all data. Data were analyzed using analysis of variance (one-way ANOVA) with Tukey’s post hoc test, considering the treatments as a fixed effect and the replicates as a random effect using 5% of significance (*P* < *0.05*) utilizing SPSS Statistics (version 29.0.1.0, IBM Corp, New York, USA).

An acceptance test was determined using the “bar chart of means” function in EyeOpenR (version 6.0.4.2, West Malling, UK) and analyzed through one-way analysis of variance (ANOVA) with Tukey’s post hoc test, where statistical differences were identified at α = 0.05 in SPSS Statistics (version 29.0.1.0, IBM Corp, New York, USA). The statistical relationship between the samples and the CATA sensory characteristics was analyzed using Cochran’s Q test and McNemar’s test, as well as Correspondence Analysis (CA) in EyeOpenR. The acceptance test and CATA results were combined in a Just About Right (JAR) analysis with the ideal in EyeOpenR.

## Data Availability

The data that support the findings of this study are available from the corresponding author upon reasonable request.
